# Concentration of low-density lipoproteins (LDL) is significantly reduced after nilotinib discontinuation

**DOI:** 10.1038/s41598-023-39057-x

**Published:** 2023-07-21

**Authors:** Ricardo Roa-Chamorro, José Manuel Puerta-Puerta, Lucía Torres-Quintero, Fernando Jaén-Águila, Pablo González-Bustos, Miguel Ángel Rodríguez-Gil, Juan Diego Mediavilla-García

**Affiliations:** 1 Vascular Risk Unit, Internal Medicine, Virgen de Las Nieves Hospital, Avenida de Las Fuerzas Armadas 2, 18014 Granada, Spain; 2 Hematology, Virgen de Las Nieves Hospital, Granada, Spain; 3 Cardiology, Virgen de Las Nieves Hospital, Granada, Spain

**Keywords:** Haematological cancer, Chronic myeloid leukaemia, Cardiovascular diseases

## Abstract

Dyslipidemia is a frequent side effect associated with nilotinib treatment. Patients with chronic myeloid leukemia (CML) under treatment with nilotinib who develop dyslipidemia have been shown to have a higher risk of presenting atherosclerotic cardiovascular disease (ACVD). Therapeutic discontinuation in selected individuals could be a strategy in order to prevent the development of ACVD. Observational study of patients with CML under nilotinib treatment. The lipid values were gathered before starting with nilotinib and after 3 months. Such values were also measured before discontinuation in patients who suspended nilotinib treatment, as well as 3 and 12 months later. 32 patients were included, 19 of them treated in monotherapy with nilotinib. The concentrations of total cholesterol and low-density lipoproteins (LDL) increased significantly after 3 months of treatment (27.29 mg/dL ± 22.88, p < 0.01). Of the total number of patients treated, 12 discontinued the treatment. LDL concentration was significantly reduced after 3 months of the nilotinib discontinuation (− 27.58 mg/dL ± 38.30, p = 0.030), remaining substantially lower after 12 months, compared to the time previous to discontinuation (− 24.58 mg/dL ± 37.31, p = 0.043). Nilotinib suspension reduces significantly LDL concentrations. These data support the strategy of therapeutic discontinuation in order to prevent future cardiovascular complications, especially in patients with prior cardiovascular risk factors.

## Introduction

The prognosis of chronic myeloid leukemia (CML) changed drastically after the introduction of tyrosine kinase inhibitors (TKI) in 2001, with the development of imatinib^1^. Subsequently, second (2GTKI) and third generation (3GTKI) TKI have been developed to be used in the front line and/or situations of intolerance or resistance to other TKI. 2GTKI have shown a faster and deeper molecular response than imatinib^2^, thus the option of discontinuing the treatment has been included in the specification sheet, provided that a series of clinical and analytical requirements are met.

A few years after the appearance of 2GTKI cardiovascular and metabolic side effects started to be notified^3^. More specifically, nilotinib has been associated with a significant increase of the lipid values in up to a 50% of the patients treated^4^, and also with peripheral arterial disease and ischemic cardiopathy, both in randomized clinical trials^2^ as well as real-life studies^5^. It has been observed that most cardiovascular events occur in patients with previous cardiovascular risk factors (CVRF) or atherosclerotic cardiovascular disease (ACVD). Thus, the current cardiovascular profile has an influence over the election of the TKI^6^. The treatment dose of nilotinib, as well as the former treatments with TKI also contribute to the pathogenesis of the ACVD^7,8^.

Dyslipidemia is one of the key factors in the development of atherosclerosis and cardiovascular disease. The atherogenesis is a progressive and complex process that takes place due to the interaction among the circulating cells and molecules, the vascular wall and the modifications in the characteristics of the bloodstream, resulting in the formation of lipid deposits in the arterial intima and the activation of the inflammatory response. Lipoproteins (LP) carry out a major role in this process, according to their number, size and proportion^9^.

LDL and other LP associated with apo B (IDL, VLDL) whose diameter is lower than 70 nm can pass freely through the intact arterial wall, interacting with the proteoglycans of the wall, thus being susceptible to remaining trapped in the extracellular matrix. Once the endothelial barrier is crossed, there can be a retention of LDL particles, especially those with a smaller size, due to their binding capacity with the proteoglycans of the extracellular matrix in the intima. This constitutes the primary event of the atherogenic process, triggering a series of modifications in the LP and local responses, including alterations of the endothelium with an increase of LDL particles permeability, monocyte recruitment (which will promote the formation of foam cells) and inflammatory changes that determine in turn a greater retention and progression of the atheromatous plaque^10^.

Given the effect that nilotinib has over the production of hypercholesterolemia, as well as its paramount role in the development of cardiovascular disease, different strategies are being implemented in order to achieve the correct evaluation and treatment of these patients^11^. Therapeutic discontinuation of nilotinib in those individuals with an adequate hematological response could represent another possibility for this population.

The objective of this study is to verify whether the dyslipidemia produced by the nilotinib treatment is reversible after the suspension of the drug.

## Materials and methods

Observational study of patients with CML under treatment with nilotinib during the last 12 months, at least. Patients belonged to the medical area of the Virgen de las Nieves Hospital in Granada, Spain. The information of the study was selected before the beginning of the TKI treatment, beginning discontinuation on the first of December of 2022.

The concentrations of total cholesterol, high-density lipoproteins (HDL), low-density lipoproteins (LDL) and triglycerides (TG) were measured before the start of the nilotinib treatment and 3 months later. In those patients eligible for discontinuation, a lipid profile was obtained before the suspension of the treatment, as well as after 3 and 12 months.

All samples were analyzed in the central laboratory of the Virgen de las Nieves University Hospital in Granada. Total cholesterol was determined in serum samples using enzymatic methods (Cholesterol Oxidase, Esterase, Peroxidase, Traceable to CDC Method). Reagent is certified to maintain traceability to the US Reference System for Cholesterol, based on the Abell-Kendall Reference Method in a network of Cholesterol Reference Method Laboratory Network, CRMLN) certified by the CDC. HDL cholesterol was determined by reaction with a specific accelerator detergent (direct method, with removal of particles and reaction with cholesterol esterase and colorimetric reading, traceable to the CDC method). The determination of LDL cholesterol was carried out by direct measurement, by colorimetric measurement using a liquid selective detergent. Triglycerides were determined using an enzymatic method (glycerol phosphate oxidase method, colorimetric) traceable to the IDMS method. The analyzer used was Alinity series C. by Abbott Diagnostics.

For the quantitative variables, their means ± standard deviation were obtained (except for age, which, due to moving away from a distribution close to a normal one, it was decided to estimate its median and interquartile range). For categorical variables, the proportions of each category were obtained. Given the normality of the distribution of cholesterol concentration and other parameters of the lipid profile, parametric tests were used. To assess the statistical significance of the differences in the concentrations of the lipid profile, Student’s t test was performed for related samples. The p threshold of statistical significance < 0.05 was taken. The software used was IBM® SPSS® Statistics v20.

This project was approved by the *Provincial Ethics Committee for Research with Medicines of Granada (CEIm/CEI)*, Spain (code 5c07f325e14f4a852ee4d0047025daf9baaa41c7). Informed consent was obtained from all patients before being included in the study. All methods were performed in accordance with the relevant guidelines and regulations.

The datasets used and analyzed during the current study are available from the corresponding author for reasonable requests.

## Results

Data were collected from 32 patients who had been treated with nilotinib at some point. At the moment of the CML diagnosis the mean age was 48 years old (IQR 35.25–59.75). 56.3% of this population were men. 19 of them were treated in monotherapy with nilotinib, whereas 13 had also been treated with other TKI (9 imatinib, 3 dasatinib and 1 with both). The initial dose of nilotinib was 300 mg in 6.3% of the patients, 600 mg in 68.8% and 800 mg. Sokal score was low in 25 patients (78.10%), intermediate in 4 patients (12.50%), and high in 3 patients (9.40%). EUTOS score was low in 29 patients (90.60%) and high in 3 patients (9.40) (Table 1).

**Table 1:** Sokal and EUTOS score at time of diagnosis.

	Whole cohort	Patients in treatment (n = 20) (%)	Discontinued treatment (n = 12) (%)
Sokal score			
High	25 (78.10)	1 (5)	2 (16.67)
Intermediate	4 (12.50)	4 (20)	0 (0)
Low	25 (78.10)	15 (75)	10 (83.33)
EUTOS score			
High	3 (9.40)	3 (15)	0 (0)
Low	29 (90.60)	17 (85)	12 (100)

SOKAL and EUTOS scores in the population treated with nilotinib.

At the moment of the data collection, the mean number of months since diagnosis was 118 (IQR 74.75–178.75). Out of the total, 20 patients (62.50%) continued with the treatment with a mean of 76.50 months (IQR 48.25–120.75).

Out of the 32 patients, 13 had suspended TKI treatment during their oncological history, although at the moment of the data collection only 12 patients (37.50%) were in a situation of therapeutic discontinuation. This was because one of them had to be reintroduced into the treatment due to failure in the discontinuation (loss of major molecular response) after 2 months. The mean duration of the nilotinib treatment in the cohort of discontinued treatment was 66.50 months (IQR 56.25–92), with a mean suspension time of 45 months (IQR 10.25–64.50). The dose of nilotinib previous to the suspension was 150 mg in 6.3% of the patients (2 patients), 300 mg in 56.3% (12 patients), 400 mg in 3.1% (1 patient) and 600 mg in 34.4% (11 patients). This information is summarized in Table 2.

**Table 2:** Median age and treatment before starting ITC and at the time of the study.

	Pre-TKI	Data collect			
Age (years)	48 (IQR 35.25–59.75)	58.50 (IQR 46.25–71)			
Time since diagnosis (months)	–	118 (IQR 74.75–178.75)	Patients in treatment 20 (62.50%)	76.50 (IQR 48.25–120.75)	
			Discontinued treatment 12 (37.50%)	Time with Nilotinib	Discontinued time
				66.50 (IQR 56.25–92)	45 (IQR 10.25–64.50)

The age of the patients is expressed in years. The time since diagnosis is expressed in months. Follow-up is divided into two groups (a) patients with continued treatment and (b) patients in therapeutic discontinuation.

*IQR* interquartile range, *TKI* tyrosine kinase inhibitor.

Table 3 outlines the data referring to the prevalence of CVRF and atherosclerotic cardiovascular disease. The information is divided in 2 groups: before initiating the TKI treatment and at the time of the study (differentiating between the cohort continuing with the nilotinib treatment at that moment and the one that had suspended it).

**Table 3:** Prevalence of CVRF and atherosclerotic cardiovascular disease in the population before starting treatment with ITC.

Time since diagnosis	PRE-TKI	Data collect	
		Nilotinib^20^	Discontinued treatment^13^
	–	118 months (IQR 74.75–178.75)	
		127 months (IQR 71.50–188)	114 months (IQR 88.25–140)
	n (%)	n (%)	n (%)
Hypertension	5 (15.62)	6 (30)	7 (58.33)
Diabetes mellitus tipe 2	3 (9.38)	6 (30)	3 (25)
Dyslipidemia	9 (31.03)	12 (60)	5 (41.67)
Smoking	4 (12.50)	2 (10)	2 (16.67)
Overweight	–	7 (35)	5 (41.67)
Obesity	–	10 (50)	5 (41.67)
Family story CVD	–	5 (25)	5 (41.67)
Ischemic heart disease	2 (6.25)	0 (0)	2 (16.67)
Cerebrovascular disease	0 (0)	2 (10)	0 (0)
Peripheral arterial disease	0 ()	3 (15)	4 (33.33)
ACVD	2 (6.25)	5 (25)	3 (33.33)

Prevalence of classic cardiovascular risk factors and atherosclerotic cardiovascular disease in the population with chronic myeloid leukemia before starting treatment with a tyrosine kinase inhibitor, before starting nilotinib and at the time of the study, differentiating between cohorts that had discontinued treatment and cohort in treatment with nilotinib. Age and time since diagnosis are represented as mean and interquartile range. Data regarding overweight and obesity before TKI treatment are not available.

*ACVD* atherosclerotic cardiovascular disease, *CVD* cardiovascular disease, *CVRF* cardiovascular risk factors, *TKI* tyrosine kinase inhibitors.

### Evolution of the lipid profile in patients treated with nilotinib

Data on the lipid profile before the TKI treatment were obtained in 29 of the 32 patients. The values of the different cholesterol fractions after 3 months of nilotinib treatment were gathered in 31 patients. The concentrations of total cholesterol, HDL, LDL and TG before starting the treatment with nilotinib and after 3 months appear in Table 4. Significant variations in the concentrations of total cholesterol, HDL and LDL were observed.

**Table 4:** Lipid profile of patients treated with nilotinib before starting treatment with a tyrosine kinase inhibitor and after 3 months of treatment with nilotinib.

	Pre-TKI (n = 29)	3 months treatment (n = 31)	Diff.	p
TC	185.28 ± 33.75	214.61 ± 32.11	29.31 ± 30.92	** < 0.01**
HDL	43.86 ± 13.40	53.94 ± 13.89	10.39 ± 11.29	** < 0.01**
LDL	109.10 ± 26.66	136.16 ± 25.90	27.29 ± 22.88	** < 0.01**
TG	155.76 ± 70.96	129.23 ± 50.83	− 26.03 ± 70.79	0.058

Lipoprotein values are expressed in milligrams per deciliter (mg/dL). Statistical significance is considered p value < 0.05.

*TC* total cholesterol, *Diff* difference expressed in absolute values and standard deviation between values of lipoprotein values before treatment and after 3 months of nilotinib, *HDL* high density lipoprotein, *LDL* Low-density lipoproteins, *TG* triglycerides.

These changes were clinically significant. Before starting with the nilotinib treatment, 9 patients (31.03%) met the definition of hypercholesterolemia (CT > 200 mg/dL and/or LDL > 160 mg/dL), although only 2 of them were being treated with hypolipidemic agents. This number increased after 3 months of treatment up to 20 subjects (64.52%). There were no modifications in the hypolipidemic treatment before starting with the TKI, compared to the visit after 3 months of starting with nilotinib.

**Figure 1:**
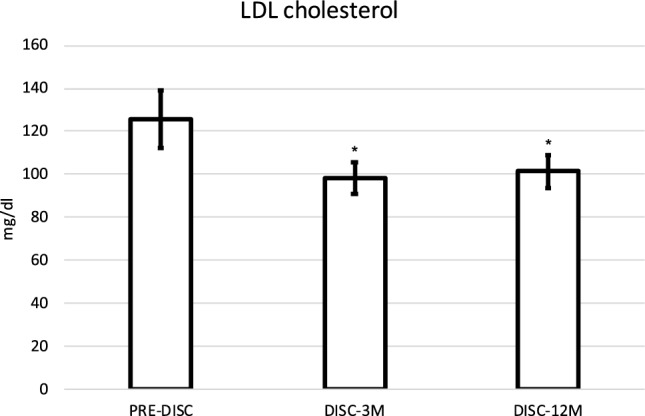
Evolution of LDL cholesterol concentrations (mg/dL) in patients with discontinuation of nilotinib). Mean ± standard error is represented. There are statistically significant differences between the concentration of LDL cholesterol before discontinuation and 3 months post-discontinuation, as well as between the concentration of LDL cholesterol before discontinuation and 12 months post-discontinuation. *p < 0.05, compared with DISC-3M. *DISC-3M* discontinuation at 3 months, *DISC-12M* discontinuation at 12 months, *PreDISC* before discontinuing nilotinib.

Regarding the 12 patients under TKI discontinuation, 7 of them (58.33%) presented dyslipidemia and all of them were being treated with statins and/or ezetimibe. There were no changes in the hypolipidemic treatment between the pre-discontinuation visit and the visits 3 and 12 months after the discontinuation. Table 5 shows the values of the different lipidic fractions before suspending the treatment with nilotinib, after 3 months and after 12 months. Significant differences were observed in the reduction of the concentration values of LDL, both after 3 months and 12 months after suspending treatment with TKI (Fig. 1).

**Table 5:** Lipid profile of patients treated with nilotinib before nilotinib discontinuation and at 3 and 12 months after treatment suspension.

	Pre-DISC (n = 12)	3M-DISC (n = 12)	12M-DISC (n = 12)	Pre-DISC—3M-DISC Diff.	p	Pre-DISC—12M-DISC Diff.	p
TC	203.42 ± 54.43	177.92 ± 24.22	183.25 ± 25.68	− 25.50 ± 46.77	0.086	− 20.18 ± 48.28	0.175
HDL	56.33 ± 15.78	53.58 ± 14.11	56.00 ± 8.48	− 2.75 ± 8.97	0.311	− 0.33 ± 10.17	0.912
LDL	125.92 ± 46.08	98.33 ± 25.46	101.33 ± 26.09	− 27.58 ± 38.30	**0.030**	− 24.58 ± 37.31	**0.043**
TG	120.83 ± 52.78	127.25 ± 38.37	129.50 ± 29.73	6.41 ± 51.34	0.673	8.67 ± 41.84	0.488

Lipoprotein values are expressed in milligrams per deciliter (mg/dL). Comparison is made with baseline levels before discontinuation. Statistical significance is considered p value < 0.05.

*DISC-3M* discontinuation at 3 months, *DISC-12M* discontinuation at 12 months, *HDL* high density lipoprotein, *Diff* difference expressed in absolute values and standard deviation), *LDL* low-density lipoproteins, *PreDISC* before discontinuing nilotinib, *TC* total cholesterol, *TG* triglycerides.

## Discussion

Treatment with TKI has improved the survival rate of patients with CML to the point of being equivalent to the general population, paired by age and gender^1^. This fact has put the spotlight on cardiovascular diseases in this population, since TKI have been associated with an increased incidence of cardiovascular disease, especially nilotinib^6^ y ponatinib^13^.

Nilotinib produces an early increase in the LDL levels, since the first month of the treatment, secondary to the higher concentration of proprotein convertase subtilisin/kexin type 9 (PCSK9), due to the mTORC1 inhibition, among other causes^14^. Besides, it has been observed that these patients present higher concentrations of LDL oxidatively modified (oxLDL) than the patients treated with imatinib and dasatinib, as demonstrated by the KIARO study in a recently published multicenter study^15^. The oxidative modification of the LDL particles increases the atherogenic potential and fosters the inflammatory response, objective in this population with the increase of inflammatory cytokines (TNF-alfa and IL-6), reduction of anti-inflammatory cytokines de (IL10) and increase in the expression of proatherogenic molecules of endothelial adhesion (E-selectin, VCAM-1, ICAM-1)^4^. Capture and accumulation of LDLox by the macrophages initiates a wide range of bioactivities that can boost the development of atherosclerotic injuries.

Recently, Caocci et al.^11^ have demonstrated that the patients with CML under treatment with nilotinib with plasmatic levels of cholesterol > 200 mg/dL and LDL > 70 mg/dL after 3 months since the start of the treatment presented a higher incidence of ACVD. Furthermore, another mechanism proposed for the development of the cardiovascular disease is the augmented prothrombotic state, which would facilitate the platelet aggregation and the generation of thrombi^16,17^.

The first clinical essay that supported the therapeutical alternative of discontinuation was published in 2016. In the French study STIM^18^, approximately 50% of the patients that suspended treatment with TKI after achieving a steady deep molecular response (DMR) for at least 2 years, maintained it after discontinuation. Besides, the vast majority of the patients who suffered a loss of response recovered it after the reintroduction of the treatment.

Treatment with 2GTKI offers greater discontinuation success, since the probability of achieving a DMP is higher with imatinib. The clinical essay ENESTfreedom^12^ showed that the duration of the nilotinib treatment regarding discontinuation is inferior compared to the one needed for the imatinib treatment. This is why the possibility of interruption has been introduced in the specification sheet of nilotinib, currently being the only drug that includes this indication^19^. The clinical requirements are a minimum treatment of 3 years and deep molecular response sustained for at least a year before the suspension of the treatment.

One of the great advantages of therapeutical discontinuation is the cessation of the exposure to the side effects of nilotinib, especially to dyslipidemia. Nevertheless, to the best of our knowledge, up to now it had not been demonstrated that the concentration values of the LDL decreased after the suspension of the therapy.

In our study, carried out with patients under nilotinib treatment (both in monotherapy and in second or third line of treatment with imatinib and dasatinib) it was observed a statistically significant increase in the values of total cholesterol and LDL after 3 months of therapy with nilotinib. This increase in cholesterol particles was also clinically important, since the prevalence of dyslipidemia rose from 31.03% before starting with nilotinib to 65.63% after 3 months of therapy.

The subjects that discontinued treatment showed a statistically significant decrease in LDL concentrations, although not in the levels of total cholesterol (however, there exists a statistical tendency). No statistically significant differences were observed between the values of the lipidic fractions 3 and 12 months after the discontinuation. Statistically, a reduction in lipid levels has not been demonstrated in patients who discontinued nilotinib. The authors consider that this fact is due to the size of our sample, therefore future studies with a larger number of patients could demonstrate our theory.

Regarding the hypolipidemic treatment of the patients under nilotinib discontinuation, 58.33% required treatment with statins and/or ezetimibe. Only one of them was under hypolipidemic treatment before starting with TKI.

Only one out of the 13 patients in our cohort who were discontinued had to reintroduce nilotinib due to failure in the hematological response. Being considered an early failure and unable to measure the lipid concentrations after 3 and 12 months, it was not included in this group of patients when the lipid concentrations were analyzed. It is noteworthy, therefore, that among the 13 patients who discontinued treatment, just one of them had to be reintroduced, resulting in a success rate of 92.30%. We do not know yet if this percentage would be equivalent to that described in the large international series^12,20,21^ when increasing the sample size.

Our study presents certain limitations. The number of patients is low since the project was carried out in a single medical center; nevertheless, it has been able to demonstrate statistically significant changes, both in the development of dyslipidemia after starting treatment with nilotinib and the reduction of the LDL concentration after TKI suspension.

## Conclusions

Nilotinib produces early dyslipidemia in a high percentage of patients treated with this drug. However, and for the first time to the best of our knowledge, it has been observed that the LDL concentrations are reduced in a statistically significant manner after therapy suspension. Thus, our data prove that the dyslipidemia produced by the nilotinib treatment is potentially reversible after the suspension of the treatment.

This fact is another reason to support the therapeutic discontinuation of TKI in patients treated for CML.

## Data Availability

The datasets used and analyzed during the current study available from the corresponding author on reasonable request.
